# Synergistic NiO‐NiCo Heterostructure Electrode for Highly Active and Durable Hydrogen Evolution in Industrial Alkaline Water Electrolysis

**DOI:** 10.1002/advs.77112

**Published:** 2026-08-15

**Authors:** Jinwoo Lee, Kyungbeen Yeom, Gaeun Hong, Yoojin Shin, Ji Seong Hyoung, Ji Eun Park, Heonjoong Lee, Taeyang Han, Yung‐Eun Sung, Jeong Won Kang, Kyoung‐Soo Kang, Hyunjoon Lee

**Affiliations:** ^1^ Hydrogen Research Department Korea Institute of Energy Research (KIER) Daejeon Republic of Korea; ^2^ Department of Chemical and Biological Engineering Korea University Seoul Republic of Korea; ^3^ School of Chemical and Biological Engineering Seoul National University Seoul Republic of Korea; ^4^ Center for Nanoparticle Research Institute for Basic Science (IBS) Seoul Republic of Korea; ^5^ Hydrogen Energy Institute Korea Institute of Energy Research (KIER) Daejeon Republic of Korea; ^6^ Graduate School of Convergence Technology and Energy Tech University of Korea Siheung Republic of Korea

**Keywords:** alkaline water electrolysis, heterostructure, hydrogen evolution reaction, nickel oxide, nickel/cobalt oxide, synergistic effect, Volmer step

## Abstract

Although its economic and environmental advantages make alkaline water electrolysis (AWE) a promising route to sustainable hydrogen production, the sluggish alkaline hydrogen evolution reaction (HER) necessitates efficient electrocatalysts to enhance AWE performance. Ni‐based materials are well‐suited for this purpose but exhibit poor alkaline HER activity because the Volmer step acts as a critical kinetic barrier restricting the overall reaction rate. To address this limitation, NiO and NiCo alloy (NiCo) are herein integrated to leverage their synergistic effects, with NiO promoting the Volmer step and NiCo providing additional catalytic sites, as confirmed by electrochemical analysis. A Ni foam–supported NiO‐NiCo electrode achieves an overpotential of 22.7 mV at 10 mA cm^−2^ under alkaline conditions, demonstrating a performance comparable with NiO and NiCo‐based electrocatalysts containing noble metals. In a single‐cell configuration, this electrode operates at 2.01 V and 1 A cm^−2^, i.e., 262 mV lower than a bare Ni‐foam electrode. Furthermore, after the on‐off durability testing, in which the current density was alternately switched between 0 and 400 mA cm^−2^ at 30 min intervals, the electrode overpotential increased by only 13 mV, indicating high stability. These results highlight the potential of NiO‐NiCo electrodes for industrial AWE.

## Graphical abstract

This study presents a NiO–NiCo heterostructure electrode that enhances water dissociation and hydrogen adsorption through synergistic interfacial interactions, verified by electrochemical and spectroscopic analyses. Its high performance in a large‐area single‐cell system under dynamic conditions demonstrates how rational non‐precious catalyst design can enable scalable hydrogen production and offer strategies for advancing sustainable electrolysis technologies.

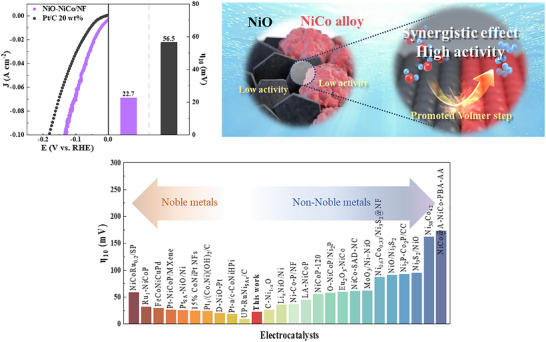

## 1 Introduction

Renewable energy–powered water electrolysis holds promise as an eco‐friendly hydrogen production method [1, 2, 3, 4, 5, 6, 7], e.g., alkaline water electrolysis (AWE) is well suited for large‐scale hydrogen production because of its low initial investment cost, high economic efficiency, and technological maturity [8, 9]. However, the activation energy of the hydrogen evolution reaction (HER) under alkaline conditions exceeds that under acidic conditions, which results in a slower reaction rate in the former case [10, 11, 12, 13]. Under alkaline conditions, the HER follows the Volmer–Heyrovsky and Volmer–Tafel pathways, and its rate is determined by the Volmer step (water dissociation and H^*^ adsorption) [14, 15, 16, 17]. Hence, high‐activity catalysts promoting the Volmer step are required to increase the overall rate of the alkaline HER [18, 19, 20, 21, 22, 23, 24, 25, 26, 27].

(1) $$H_{2}O+e^{-}\;\to \;H^{*}+{\mathrm{OH}}^{-}\;\left[\mathrm{Volmer},\;120\;\mathrm{mV}\;{\mathrm{dec}}^{-1}\right]$$

(2) $$H_{2}O+e^{-}+H^{*}\;\to \;H_{2}+{\mathrm{OH}}^{-}\;\left[\mathrm{Heyrovsky},\;40\;\mathrm{mV}\;{\mathrm{dec}}^{-1}\right]$$

(3) $$H^{*}+H^{*}\;\to \;H_{2}\;\left[\mathrm{Tafel},\;30\;\mathrm{mV}\;{\mathrm{dec}}^{-1}\right]$$

Noble metals (e.g., Pt, Ru) can be introduced to lower the energy barrier of the water dissociation step and increase conductivity to overcome these limitations and enhance the HER performance, although the scarcity and high cost of these metals pose economic constraints. Thus, transition metal (TM)‐based materials can exhibit economically viable and high HER activities if the Volmer step is promoted without the use of noble metals. Metal oxides are known to promote the Volmer step; e.g., NiO facilitates this step because of the preferential attachment of OH^−^ ions to Ni sites with unstable d‐orbitals, which favors H adsorption on adjacent transition‐metal sites [28, 29]. However, pure‐NiO electrodes have limited HER activities because of the insulating nature of NiO and insufficient number of H adsorption sites. Furthermore, based on the Sabatier principle, single‐component catalysts cannot simultaneously optimize the water dissociation and binding energies of H^*^ and ^*^OH intermediates [30]. Heterostructure has emerged as an effective strategy to overcome these limitations by modulating the interfacial electronic structure and optimizing the adsorption behavior of reaction intermediates through synergistic interactions between different components. Xiaobo Zheng et al. reported that the Pt/LiCoO_2_ heterostructure exhibits interfacial electronic interactions, enabling active‐center transfer between Pt and LiCoO_2_ and thereby promoting overall water‐splitting activity [31]. Guoqiang Zhao et al. also demonstrated that an Ir/Ni(OH)_2_ heterostructure enables strong electronic interactions at the heterointerface, which stabilizes active Ir(V) species and promotes the adsorption behavior of OER intermediates [32]. Accordingly, extensive efforts have been made to engineer NiO‐based electrodes by integrating various transition metals and constructing heterogeneous interfaces, which exhibit high HER activity and overall electrochemical performance [33, 34, 35, 36, 37, 38, 39, 40, 41, 42]. For instance, Dai et al. demonstrated that a NiO/Ni heterostructure on carbon nanotubes exhibited Pt‐like HER activity, which is attributed to a synergistic interfacial effect where NiO sites preferentially adsorb OH^*^, and nearby Ni facilitates H adsorption, thereby facilitating efficient water adsorption and dissociation and accelerating the Volmer step [28]. Zhang et al. also reported that the Ni_3_S_2_/NiO heterostructure provides synergistic interfacial sites, where interfacial Ni facilitates water dissociation and OH^*^ adsorption, while interfacial S sites promote H^*^ adsorption and H_2_ evolution, enabling high HER activity [41]. Such synergistic coupling significantly lowers the energy barrier of the Volmer step while maintaining favorable energetics for the Heyrovsky or Tafel step, leading to enhanced hydrogen evolution kinetics and improved catalytic durability in alkaline media. However, the HER performance of heterostructure materials is still far from industrial expectations.

To overcome these limitations, we present, for the first time, an electrode with surface‐exposed NiO and NiCo (NiO‐NiCo), leveraging the complementary advantages of the two materials (OH^−^ adsorption properties of NiO and multiple and synergistic active sites of NiCo) to lower the energy barrier of the Volmer step and thus enhance HER activity. NiCo was selected because of its high catalytic efficiency and adaptability to the alkaline HER [43, 44, 45]. NiO/Ni was synthesized through a hydrothermal process followed by annealing, and NiCo alloy was electrodeposited onto areas where NiO/Ni was not formed by exploiting the insulating properties of this oxide. The NiO‐NiCo electrode exhibited a heterostructure with coexisting NiO and NiCo. The electrodeposition time was systematically controlled to achieve an appropriate interfacial exposure between NiO and NiCo. Since NiO and NiCo play distinct roles during the alkaline HER and their synergistic activity originates from electronic modulation at the heterojunction, establishing optimal interfacial exposure is essential for maximizing the synergistic effect of the heterostructure. The comparative electrochemical analysis of the NiO‐NiCo, NiO‐only, and NiCo‐only electrodes confirmed the synergistic effects of the two materials and showed that this synergy enhanced the HER performance by promoting the Volmer step. The overpotential of Ni foam (NF)‐supported NiO‐NiCo (22.7 mV at 10 mA cm^−2^) was comparable with that of noble metal–based electrodes. In an industrial applicability test, performed in a single‐cell configuration with NiO‐NiCo on NF (surface area = 59.4 cm^2,^ diameter = 87 mm) as the cathode, the operating voltage at a current density of 1 A cm^−2^ (2.01 V) was 262 mV lower than that observed for bare NF. After a dynamic operational test under intermittent conditions, the overpotential at 1 A cm^−2^ increased by only 13 mV, demonstrating the high durability and industrial application potential of NiO‐NiCo electrodes.

## 2 Results and Discussion

### 2.1 Synthesis and Characterization of NiO‐NiCo

**FIGURE 1:**
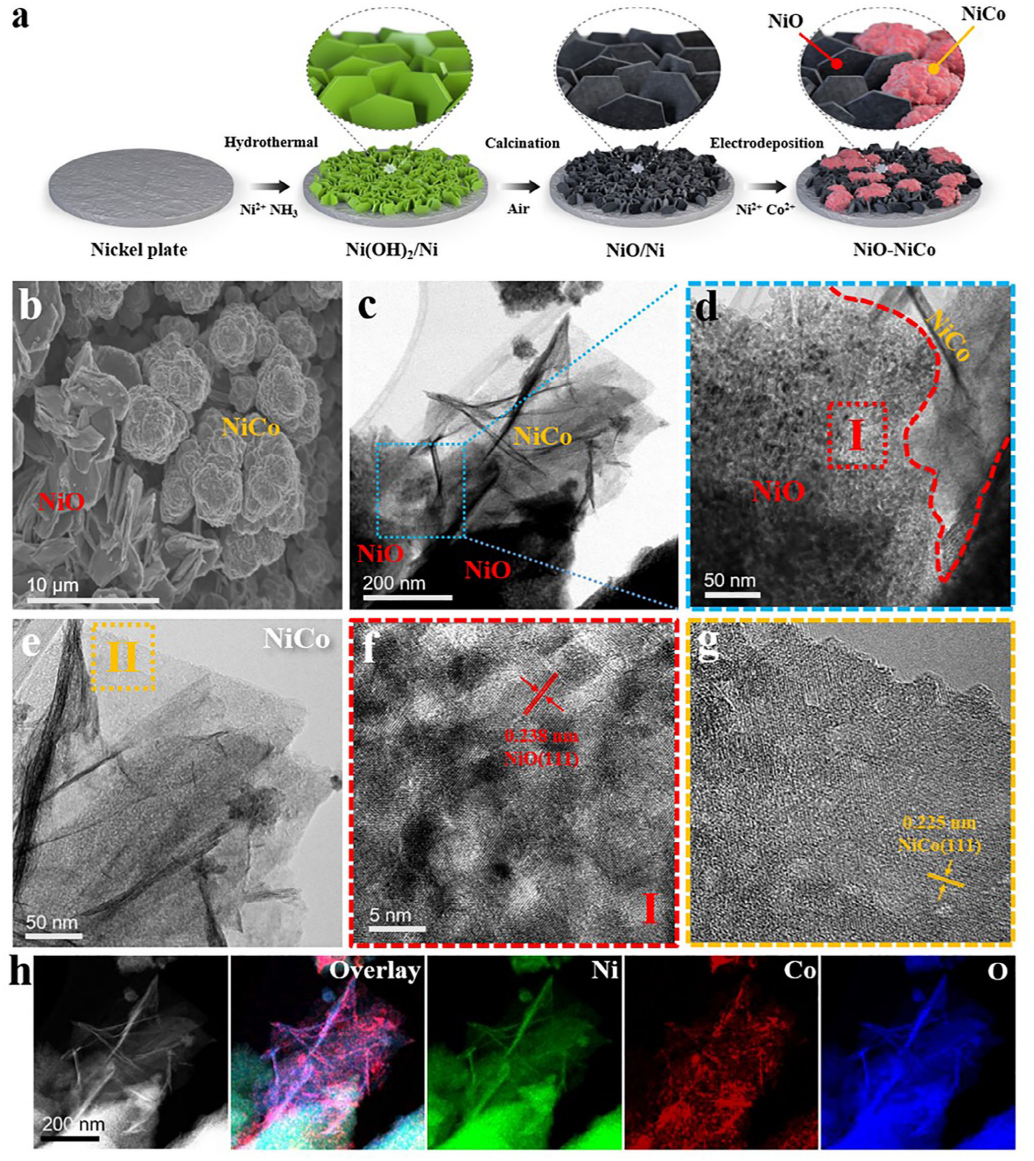
(a) Schematic synthesis, (b) scanning electron microscopy image, and (c) transmission electron microscopy (TEM) image of NiO‐NiCo. (d) Magnified TEM image of the region with the NiO–NiCo interface in NiO‐NiCo (blue rectangle–enclosed area in (c)). (e) Magnified TEM image of the NiCo part in NiO‐NiCo. (f,g) High‐resolution TEM images of regions I (red rectangle–enclosed area in (d)) and II (yellow rectangle–enclosed area in (e)) used to measure the lattice fringe spacings of NiO and NiCo in NiO‐NiCo. (h) Scanning TEM–energy‐dispersive X‐ray spectroscopy elemental mapping of NiO‐NiCo.

**FIGURE 2:**
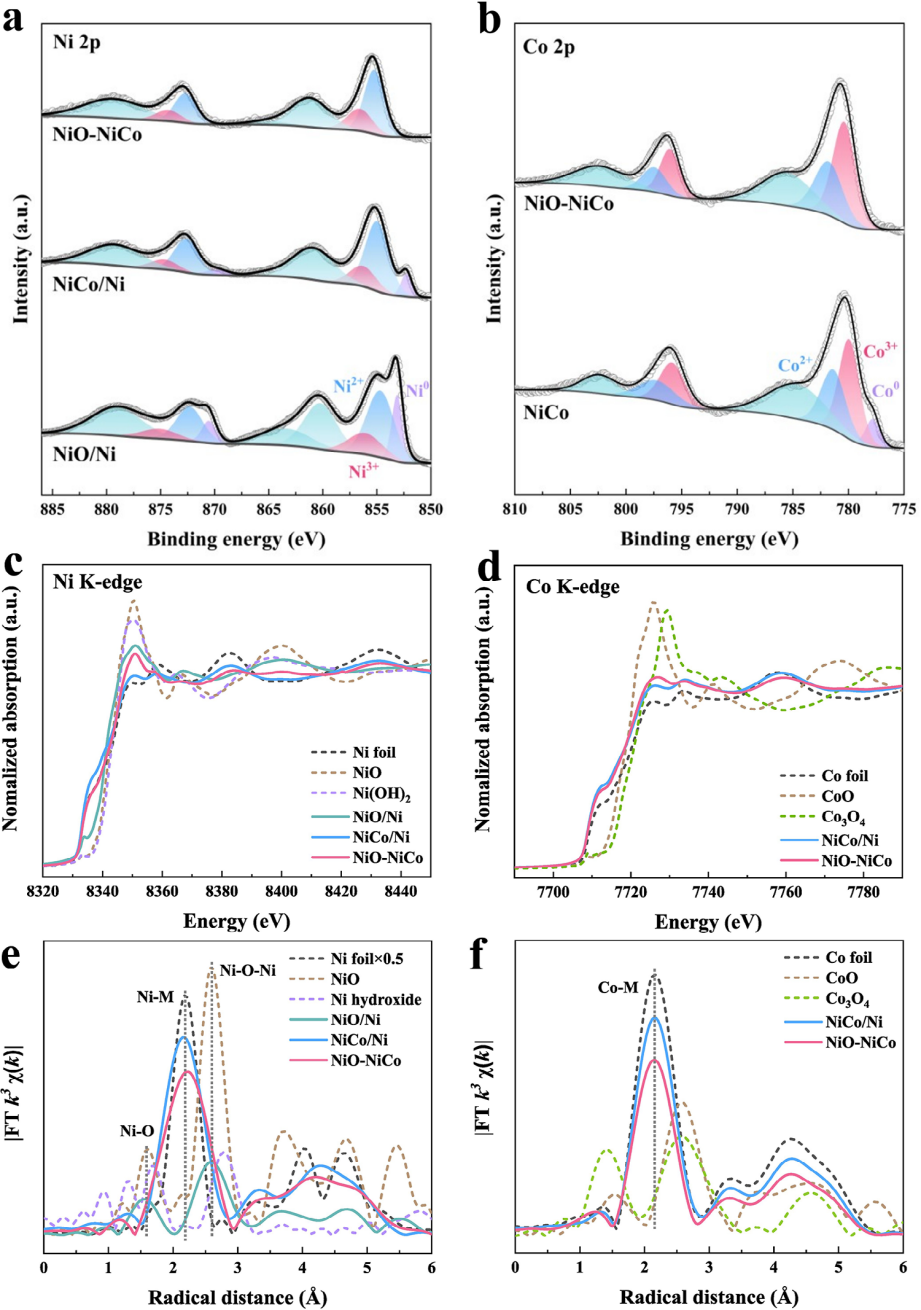
(a) Ni 2p, and (b) Co 2p XPS spectra of NiO/Ni, NiCo/Ni, and NiO‐NiCo. (c) Normalized Ni K‐edge XANES spectra and (d) Co K‐edge XANES spectra of NiO/Ni, NiCo/Ni, NiO‐NiCo, and references. (e) Corresponding Ni K‐edge EXAFS spectra and (f) Co K‐edge EXAFS spectra of NiO/Ni, NiCo/Ni, NiO‐NiCo and references.

The NiO‐NiCo electrode was synthesized in three steps (Figure 1a) using a flat Ni plate (Figure S1a) as a substrate to focus on catalyst properties. Ni(OH)_2_/Ni comprised plate‐like Ni(OH)_2_ particles (Figure S1b), and the corresponding XRD pattern (Figure S2) featured the (001), (100), (011), (012), (110), (111), (103), and (021) peaks of Ni(OH)_2_ (JCPDS No. 14–0117) at 19.2°, 33°, 38.5°, 52°, 58.9°, 62.6°, 70.4°, and 72.6°, respectively, and those of the Ni substrate (JCPDS No. 04–0850) at 44.5°, 51.8°, and 76.3°. NiO/Ni was synthesized by calcining Ni(OH)_2_ at 400°C in an air atmosphere. The rapid weight loss of Ni(OH)_2_ above 300°C indicated that the employed calcination temperature of 400°C was sufficient for the decomposition of Ni(OH)_2_ to NiO (Figure S3). The XRD pattern of NiO/Ni (Figure S4) showed the (111), (200), and (220) peaks of NiO (JCPDS No. 47–1049) at 37.2°, 43.2°, and 62.8°, respectively. The absence of Ni(OH)_2_ peaks confirmed the complete decomposition of this hydroxide into NiO, which retained the plate‐like structure of its precursor (Figure S5). The TEM imaging of NiO/Ni (Figure S6) revealed stacked nanosheet structures forming a plate‐like structure. The NiO‐NiCo electrode was fabricated by exploiting the insulating properties of NiO to selectively electrodeposit NiCo onto areas where NiO was not formed (Figure 1a). The plate‐like structure of NiO/Ni was retained after electroplating, and the deposited NiCo comprised cauliflower‐shaped particles (Figure 1b). EDS‐based elemental mapping showed that these particles mainly contained Co, indicating the coexistence of NiO and NiCo on the electrode surface (Figure S7). The TEM analysis of NiO‐NiCo revealed regions where NiO and NiCo were in contact (Figure 1c,d) and areas where they existed independently (Figure S8). The TEM image in Figure 1c shows the coexistence of NiO and NiCo, while the corresponding enlarged view in Figure 1d reveals that NiO and NiCo are adjacent but maintain distinct structural characteristics. NiO retained its thin nanosheet structure after electrodeposition (Figure S8a–c). NiCo/Ni had an almost monolayer nanosheet structure (Figure 1e), while NiCo/Ni also formed a layered nanosheet structure but (unlike NiO) comprised cauliflower‐shaped particles (Figure S8d–f). The fine structures of NiO/Ni and NiCo/Ni were characterized using high‐resolution transmission electron microscopy. Figure 1f,g show the high‐resolution TEM images of region I (red rectangle–enclosed area) in Figure 1d and region II (yellow rectangle–enclosed area) in Figure 1e, respectively. The lattice fringes of the NiO had a spacing of 0.238 nm corresponding to the (111) plane [46], while those of the NiCo alloy had a spacing of 0.225 nm corresponding to the plane [47]. These observations indicated that NiO and NiCo were adjacent to each other but possessed distinct structural features, demonstrating that NiO/Ni was not influenced by electrodeposition, as confirmed by EDS‐coupled scanning TEM. In the elemental mappings of adjacent NiO and NiCo, Co was predominantly present in the NiCo regions and not in NiO, which demonstrated that NiO was minimally affected by electrodeposition (Figure 1h). These findings were consistent with the SEM‐EDS mappings in Figure S7b. The precise effect of electrodeposition was confirmed through EDS analysis of electrodes where NiO and NiCo were individually present in NiO‐NiCo (Figure S9). The Co content of NiCo exceeded 10.1 at.%, while that of NiO was below 1 at.%, which indicated that NiO was hardly influenced by deposition. These results suggested that owing to the insulating nature of NiO, deposition predominantly occurred in regions where NiO was absent to afford a structure characterized by both the copresence and separate existence of NiO and NiCo. To determine the Ni and Co contents of NiCo/Ni, EDS analysis was performed at three different locations of the NiCo part (Figure S10). The Co/Ni ratio (average 0.568) indicated the uniform formation of NiCo and was close to that (0.6) of the precursor solution added during electrodeposition. Given that NiO‐NiCo was expected to exhibit the characteristics of both NiO and NiCo because of their coexistence, a NiCo‐only electrode was fabricated for comparative analysis by electrodeposition on a Ni plate under the conditions used for NiO‐NiCo, NiCo/Ni was synthesized by constant voltage electrodeposition at ‐7.4 V for 125 s, the voltage used for electrodepositing NiO‐NiCo (Figure S11). The SEM imaging of NiCo/Ni showed a cauliflower morphology similar to that of NiCo/Ni in NiO‐NiCo (Figure S11a), while TEM imaging revealed an alloy structure identical to that of NiO‐NiCo (Figure S11b–e). EDS analysis showed uniform Ni and Co distributions (Figure S11f) and revealed that the elemental composition of NiCo (Figure S11g) was almost identical to that of NiCo coexisting with NiO (Figure S9c). When the electrodeposition time was insufficient (30 s), only a scarce amount of NiCo was formed, resulting in inadequate heterointerface formation (Figure S12a,c). In contrast, excessive electrodeposition time led to excessive NiCo growth (600 s), which covered the active sites of NiO rather than forming a heterostructure (Figure S12b,d). Given the limitations of SEM and TEM as localized analysis methods, additional analyses were conducted for a more precise understanding of the properties of NiO‐NiCo. The XRD pattern of NiO‐NiCo showed NiO peaks, indicating the presence of NiO on the surface (Figure S13). However, no peaks related to NiCo were observed for NiCo‐containing electrodes, which was ascribed to the strong influence of the Ni substrate and lower crystallinity of NiCo compared with that of other materials [48]. Therefore, we probed the chemical structure and surface chemical states of NiCo using Raman spectroscopy, which is suitable for analyzing structures, and XPS, which minimizes substrate interference and is sensitive to surface composition (Figure 2a,b, and Figure S14). The Raman spectra of Ni(OH)_2_/Ni (Figure S15) showed Ni(OH)_2_ peaks at 314, 452.8, and 3583.9 cm^−1^ [49]. These peaks were absent in the spectrum of NiO/Ni (Figure S15), which featured signals at 401.5 and 459.9 cm^−1^ are attributed to transverse optical (TO) phonon modes, while the peak at 495.0 cm^−1^ corresponds to one‐phonon and one‐magnon (1P + 1 M) mode and the peak at 1064.2 cm^−1^ corresponds to the two‐phonon (2P) mode of NiO [50]. The spectrum of NiCo/Ni (Figure S14) featured peaks at 459.9 and 528.3 cm^−1^, which are attributed to the TO_2_ phonon and longitudinal optical (LO) phonon modes, derived from the surface oxidation of metallic NiCo alloys [43, 51, 52, 53]. As expected, both NiO‐ and NiCo‐related peaks were observed for NiO‐NiCo, in agreement with the results of SEM and TEM analyses, indicating the coexistence of NiO and NiCo on the electrode surface. XPS analysis (Figure S16) revealed that the Ni^0^ 2p_3/2_ peak of the Ni plate at 852.6 eV was not observed for Ni(OH)_2_/Ni [54]. The Ni 2p spectra of the latter featured only the Ni^2+^ 2p_3/2_ peak of Ni(OH)_2_ at 855.4 eV, indicating that, in contrast to the XRD analysis, the synthesized electrode exhibited its own properties without influence from the Ni substrate. The Ni 2p spectra of NiO/Ni showed a Ni^0^ 2p_3/2_ peak at 853.1 eV, attributed to the exposed Ni plate surface after calcination, along with a Ni^2+^ 2p_3/2_ peak at 854.7 eV, with the binding energy reduction ascribed to the combination of Ni^2+^ with oxygen. During calcination in an oxidizing atmosphere, Ni^2+^ was oxidized to Ni^3+^, and a Ni^3+^ 2p_3/2_ peak at 856.1 eV was observed (Figure 2a) [55, 56]. The Ni 2p spectra of NiCo/Ni showed Ni^0^, Ni^2+^ 2p_3/2_, and Ni^3+^ 2p_3/2_ peaks at 852.3, 855.0, and 856.3 eV, respectively [43, 44, 51]. The Ni^0^ peak is attributed to the metallic features of the NiCo alloy, while the formation of the Ni^2+^ and Ni^3+^ peaks arises from the surface oxidation of the metallic NiCo alloy [43, 51]. Such surface oxidation is commonly observed for alloy materials upon exposure to air, resulting in the formation of a thin surface oxide layer. Consequently, XPS spectra generally exhibit both metallic species and oxidized species, because XPS is a surface‐sensitive technique that probes only a few nanometers of the sample. For the NiO‐NiCo electrode, the Ni^2+^ and Ni^3+^ 2p_3/2_ peaks are observed at 855.2 and 856.5 eV, respectively. The disappearance of the Ni^0^ peak of NiCo is indicative of electron transfer from NiCo to NiO [57, 58]. Additionally, the two peaks observed for NiCo were also detected for NiO‐NiCo. These findings indicated the coexistence of NiO and NiCo. Moreover, the Ni^3+^/Ni^2+^ ratio of the NiO‐NiCo is intermediate between those of NiO/Ni and NiCo/Ni, suggesting the interfacial electron transfer and modulation of the local electronic structure at the NiO‐NiCo heterointerface (Table S1). The Co 2p spectra of NiCo/Ni also revealed the metallic Co^0^ state at 777.8 eV, along with Co^2+^ and Co^3+^ 2p_3/2_ peaks at 781.4 and 779.9 eV, indicating partial oxidation of Co (Figure 2b) [41, 47, 53]. In contrast, the Co 2p spectra of NiO‐NiCo exhibited only Co^2+^ and Co^3+^ 2p_3/2_ peaks at 781.8 and 780.4 eV, respectively. Moreover, the Co^3+^/Co^2+^ ratio of NiO‐NiCo is slightly increased compared with NiCo/Ni (Table S2). From these results, interfacial NiCo becomes slightly oxidized through interaction with O anions, accompanied by electron transfer from the O anions to the neighboring Ni in NiO [57]. To further support the coexistence of the NiO and NiCo phases, we additionally included Ni L‐edge and Co L‐edge spectra of the samples (Figure S17). X‐ray absorption fine structure (XAFS) and extended X‐ray absorption fine structure (EXAFS) spectroscopy are employed to investigate the valence and coordination environment of the NiO/Ni, NiCo/Ni, NiO‐NiCo, and references (Figure 2c–f). The Ni K‐edge XANES spectra for NiCo/Ni is similar to that of standard Ni foil, and significantly differ from the NiO reference, suggesting that NiCo/Ni predominantly exists in a metallic phase due to NiCo alloys [43, 44]. In contrast, the Ni K‐edge XANES spectra for NiO appear between those of the standard Ni foil and NiO reference, indicating the main phase of the electrode is NiO [59]. Similarly, the Co K‐edge XANES spectra, both NiCo/Ni and NiO‐NiCo exhibited similar profiles to that of the standard Co foil, indicating the oxidation states of Co were zero. Additionally, the enhanced white line intensity and subtle spectral differences observed in NiCo/Ni and NiO–NiCo suggest modifications in the local electronic structure, reflecting strong electronic interactions between Ni and Co that are intrinsic to alloyed materials [43]. Notably, the NiO–NiCo exhibits intermediate features between those of NiO/Ni and NiCo/Ni, reflecting the co‐existence of NiO and NiCo. The Ni K‐edge EXAFS spectra of NiCo/Ni and NiO‐NiCo showed a peak at ∼2.2 Å, attributed to the Ni─M (M═Co, Ni) bonds. In contrast, the Ni K‐edge EXAFS spectra of NiO display peaks at ∼1.6 Å and ∼2.6 Å, which are assigned to the Ni─O and Ni─O─M bonds, respectively, which are characteristic of the NiO phase (Figure 2e) [43, 44]. Similarly, the Co K‐edge EXAFS spectra of NiCo/Ni and NiO‐NiCo also exhibited a peak similar to that of standard Co foil at approximately ∼2.2 Å, corresponding to the Co─M (M═Co, Ni) bonds (Figure 2f) [43, 59]. The lower intensity of the Co─M and Ni─M coordination shells in NiO‐NiCo compared to NiCo/Ni implies a reduced coordination number, which can be attributed to the co‐existence of NiO and NiCo. These results indicate that the electronic structure of NiO‐NiCo is intermediate between NiO/Ni and NiCo/Ni, consistent with the XPS results. Based on these results, the NiO‐NiCo heterostructure promotes interfacial electron transfer from NiCo to the adjacent NiO. NiCo becomes slightly oxidized, which optimizes the metal‐hydrogen bond binding energy, while the increased charge density on Ni atoms in NiO enhances the adsorption and dissociation of H_2_O molecules [37, 57]. It has been reported that interfacial electronic interactions between adjacent metal species can regulate the electronic structure of active sites, thereby optimizing the adsorption energies of reaction intermediates and accelerating electrocatalytic kinetics [60, 61]. Similarly, the Ni interface in the NiO‐NiCo heterostructure promotes interfacial charge transfer between NiO and NiCo, leading to synergistic effects that facilitate both the Volmer step and hydrogen evolution. The increased electron density around Ni atoms in NiO is expected to modulate the Ni 3d electronic structure, thereby providing more favorable active sites for H_2_O adsorption and significantly lowering the energy barrier for H─OH bond cleavage during alkaline HER. Such electronic modulation facilitates the Volmer step by promoting water activation and dissociation, leading to the generation of adsorbed hydrogen for the subsequent hydrogen evolution process [37,^62^]. Moreover, Di Gao et al. reported that the NiCo alloy exhibits strong hydrogen adsorption, and they further demonstrated that the W incorporation modulates the electronic structure of NiCo alloy, optimizes the metal–H bond strength by tuning the hydrogen adsorption energy, and consequently accelerates the HER [57]. Similarly, the interfacial electronic interaction between NiO and NiCo is expected to modulate the electronic structure of NiCo, thereby optimizing the M─H bond strength.

### 2.2 HER Performance of NiO‐NiCo

**FIGURE 3:**
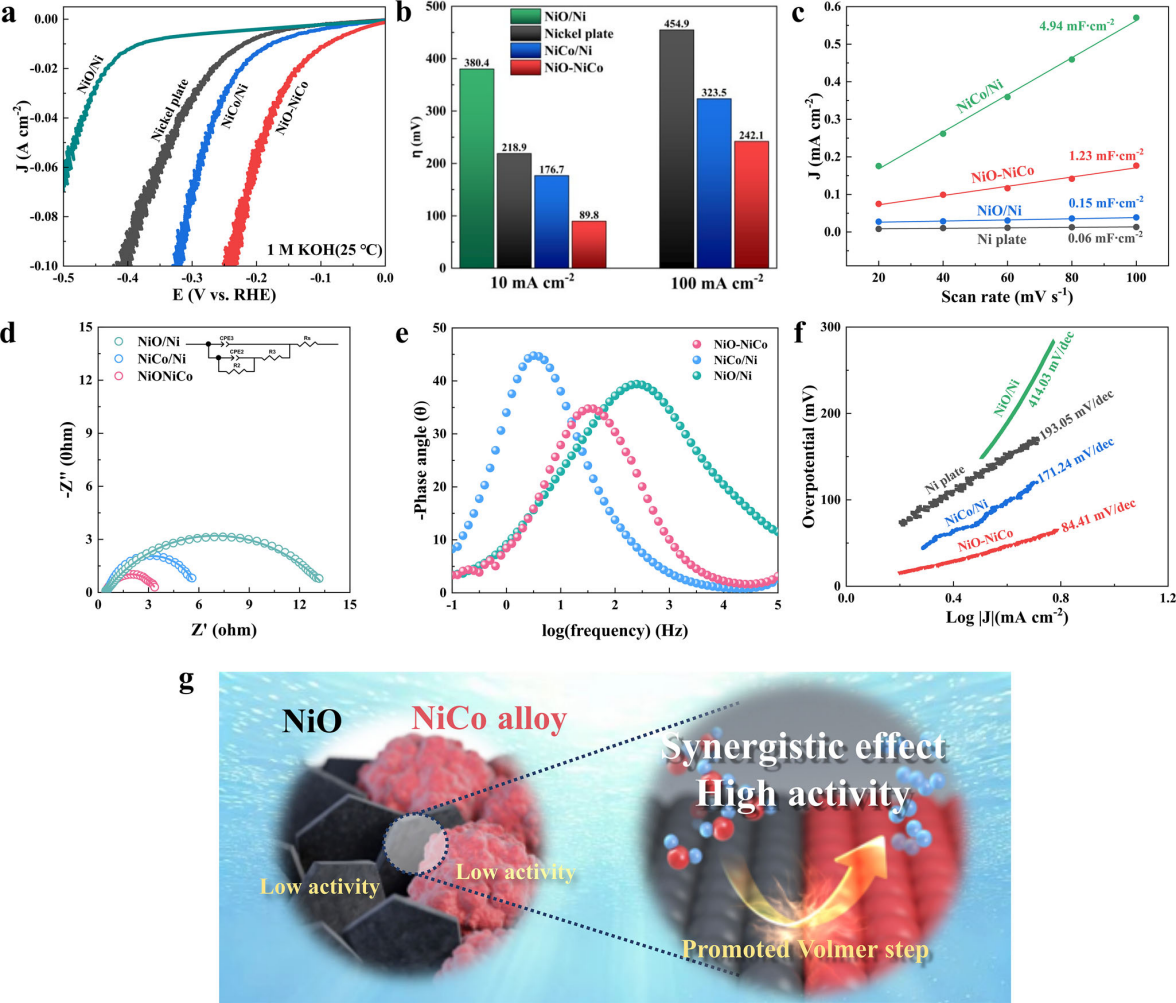
(a) Linear sweep voltammetry (LSV) curves. (b) Overpotentials at 10 and 100 mA cm^−2^. (c) Charging current density differences at different scan rates used to obtain the electrochemically active surface areas of the Ni plate, NiO/Ni, NiCo/Ni, and NiO‐NiCo. (d) Nyquist plots and (e) Bode plots of the EIS spectra for the HER on NiO/Ni, NiCo/Ni, and NiO‐NiCo in 1 M KOH. (f) Tafel slopes of Ni plate, NiO/Ni, NiCo/Ni, and NiO‐NiCo. (g) Schematic of the synergistic effect in NiO‐NiCo.

The expected electrochemical performance enhancement due to the coexistence of NiO and NiCo was verified by evaluating the HER performance of NiO‐NiCo under alkaline conditions using LSV (Figure 3a) and overpotentials at current densities of 10 and 100 mA cm^−2^ (Figure 3b). NiO/Ni required a higher overpotential at 10 mA cm^−2^ (380.4 mV) than the bare Ni plate (218.9 mV @ 10 mA cm^−2^, 454.9 mV @ 100 mA cm^−2^), as NiO had almost no HER activity because of its insulating nature and lack of hydrogen adsorption sites [28]. The NiCo/Ni electrode outperformed the bare Ni plate (176.7 mV @ 10 mA cm^−2^, 323.5 mV @ 100 mA cm^−2^), which was ascribed to the cauliflower structure of NiCo providing abundant active sites because of its large surface area. NiO‐NiCo showed an activity (89.8 mV @ 10 mA cm^−2^, 242.1 mV @ 100 mA cm^−2^) superior to (and not intermediate between) those of NiO/Ni and NiCo/Ni, showing overpotentials at 10 and 100 mA cm^−2^ that were 86.9 and 81.4 mV lower than those of NiCo/Ni, respectively. The synergistic effect of NiO and NiCo was further supported by the results of ECSA measurements (Figure 3c and Figure S19). The *C*_dl_ and ECSA of NiO‐NiCo (1.23 mF cm^−2^ and 30.75 cm^2^, respectively) were intermediate between those of NiO/Ni (0.15 mF cm^−2^, 3.75 cm^2^) and NiCo/Ni (4.94 mF cm^−2^, 123.5 cm^2^), unlike the previously discussed electrochemical performance metrics. However, despite having a smaller ECSA than NiCo/Ni, NiO‐NiCo exhibited a superior electrochemical performance, indicating a substantial increase in specific activity, which confirmed the synergy between NiO and NiCo (Figure 3c). The electrochemical impedance spectroscopy (EIS) was conducted to gain deeper insight into the HER kinetics of the heterostructure (Figure 3d,e). The Nyquist plots were simulated by using an equivalent circuit model composed of the electrolyte resistance (R_s_), reaction intermediate accumulation (R_2_ and constant phase element (CPE_2_)) associated with the Volmer step in the low‐frequency region, and interfacial charge transfer (R_3_ and CPE_3_) corresponding to the Heyrovsky step in the middle‐frequency region (Figure S18a) [63]. According to the circuit fitting results shown in Figure S18b, NiCo/Ni exhibits a much lower R_3_ than NiO/Ni, which is associated with the Heyrovsky step. This is attributed to the high electrical conductivity of the NiCo alloy [43, 64]. Moreover, NiO‐NiCo presents lower R_2_ and R_3_ compared to NiO/Ni and NiCo/Ni, corresponding to the Volmer and the Heyrovsky steps, respectively, resulting from the synergistic effect of the heterostructure. This result is further supported by the Bode plot analysis, where NiCo/Ni exhibits its phase maximum in the low‐frequency region, indicating that the Volmer step is the rate‐determining step (RDS). In comparison, the maximum phase of NiO‐NiCo is in the middle frequency region, as NiO facilitates the H_2_O adsorption and dissociation during the Volmer step, thereby accelerating the subsequent reaction [65, 66]. Consistent with the EIS results, Tafel slope analysis confirms the accelerated HER kinetics on NiO‐NiCo. The HER kinetics were analyzed using Tafel plots derived from polarization curves to determine the synergistic effects of NiO and NiCo (Figure 3f). Generally, lower Tafel slopes indicate higher catalyst efficiencies, which allows RDS identification. NiO‐NiCo (84.41 mV dec^−1^) exhibited a lower Tafel slope than NiCo/Ni (171.24 mV dec^−1^) and NiO/Ni (414.03 mV dec^−1^) and was therefore concluded to achieve a higher HER rate, whereas NiO/Ni exhibited a higher slope than the Ni plate (193.05 mV dec^−1^), which indicated a very low rate. Consistent with the results of previous electrochemical performance evaluation, NiO‐NiCo displayed a notably enhanced reaction rate exceeding those observed individually for NiO and NiCo, while NiO/Ni demonstrated a low HER activity because of its insulating properties and lack of H adsorption sites. In alkaline electrolytes, the HER comprises Volmer (120 mV dec^−1^), Heyrovsky (40 mV dec^−1^), and Tafel (30 mV dec^−1^) steps, with the Volmer step generally determining the overall rate. Therefore, the acceleration of the Volmer step improves electrode performance [18, 19, 20, 21]. NiCo/Ni exhibited a Tafel slope higher than the Volmer‐step value of 120 mV dec^−1^, whereas the reverse was true for NiO‐NiCo. This indicated that the introduction of NiO promoted the Volmer step, which was hindered by the inherent alloying materials, thereby enhancing performance. These results suggested that the coexistence of NiO and NiCo on the surface resulted in a synergistic effect, leading to fast charge transfer and superior performance (Figure 3g).

**FIGURE 4:**
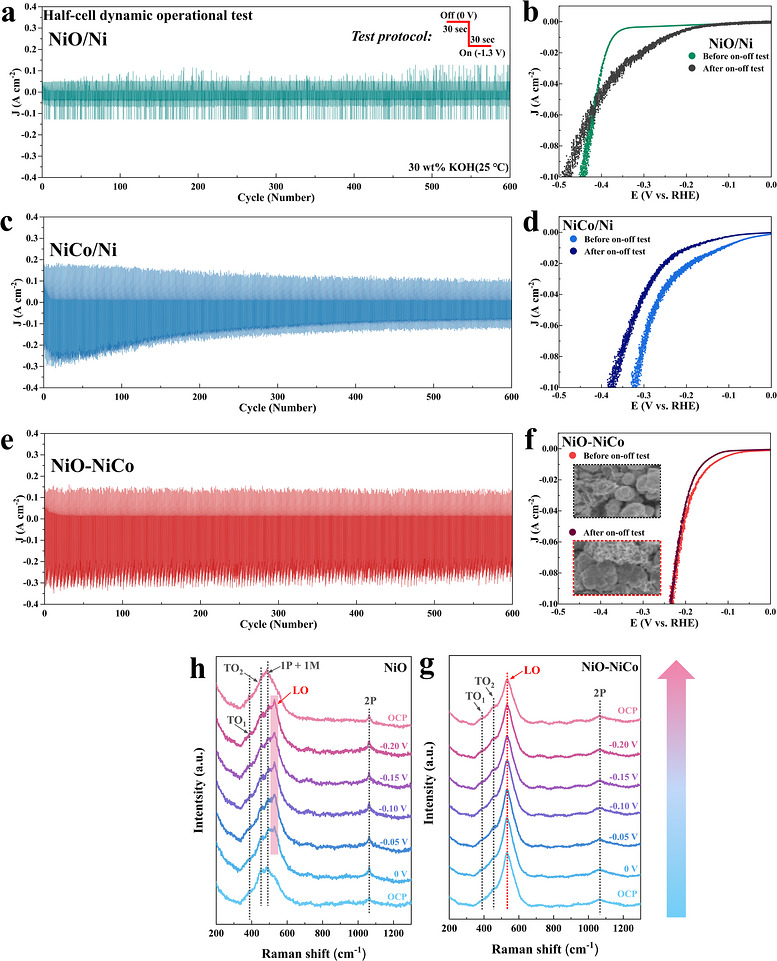
(a,c,e) Results of the half‐cell dynamic operation (on‐off) test and (b,d,f) LSV curves recorded before and after this test for (a,b) NiO/Ni, (c,d) NiCo/Ni, and (e,f) NiO‐NiCo. In situ Raman spectra of (g) NiO/Ni and (h) NiO‐NiCo for HER.

The durability of NiO‐NiCo was evaluated under dynamic operational conditions simulating those of AWE (Figure 4a–f). During the test, current densities above 0 mA cm^−2^ during the off periods indicated the occurrence of reverse current due to sudden voltage changes, suggesting the ease of HER performance degradation under the employed harsh conditions. The durability testing of NiO/Ni showed that it maintained a low‐activity state (Figure 4a). The differences in the LSV curves before (NiO: 371 mV @ 10 mA cm^−2^, 442 mV @ 100 mA cm^−2^) and after (NiO: 246.5 mV @ 10 mA cm^−2^, 483.1 mV @ 100 mA cm^−2^) on‐off testing (Figure 4b) were attributed to the collapse of the NiO structure through reduction and resulting Ni substrate exposure [67]. SEM imaging confirmed the collapse of the NiO plate‐like structure (Figure S20a), and EDS analysis showed that O from NiO was not evenly distributed across the electrode but was found only in certain areas, indicating that most of the NiO was deformed (Figure S20b). NiCo/Ni showed enhanced performance up to 20 cycles but suffered from performance degradation after the 21st cycle (Figure 4c). The overpotentials after the on‐off test (192.8 mV @ 10 mA cm^−2^, 376.2 mV @ 100 mA cm^−2^) exceeded those before the test (114.4 mV @ 10 mA cm^−2^, 321.8 mV @ 100 mA cm^−2^), indicating performance degradation (Figure 4d) due to the structural degradation of NiCo (Figures S21a,b). In contrast, NiO‐NiCo maintained its performance over 600 cycles (Figure 4e). The overpotentials before (136.9 mV @ 10 mA cm^−2^, 232.5 mV @ 100 mA cm^−2^) and after the test (158.6 mV @ 10 mA cm^−2^, 233.8 mV @ 100 mA cm^−2^) were similar, indicating stable performance (Figure 4f). SEM imaging indicated that the structural collapse was less pronounced than observed for the other electrodes, revealing that the coexistence of NiO and NiCo was maintained (Figure S22a,b). Furthermore, the NiO‐NiCo electrode exhibited stable performance at 100 mA cm^−2^ for 100 h (Figure S23). TEM and XPS analyses further confirmed that the overall morphology and oxidation states of Ni and Co were well preserved after the long‐term test, demonstrating the robust stability of the NiO‐NiCo heterostructure electrode (Figures S24a, S25a,b). Although further studies are needed to determine the exact cause, the NiO‐NiCo electrode showed high electrochemical durability and structural stability even under fluctuating loading conditions. In situ Raman spectroscopy was conducted to understand the structural robustness of NiO‐NiCo during HER (Figure 4g,h). Shell‐isolated nanoparticle‐enhanced Raman spectroscopy (SHINER) was employed to amplify the Raman signal [68]. As shown in Figure 4g, at OCP, a peak at 485.9 cm^−1^ is observed, which is assigned to the 1P + 1 M mode of NiO. With the applied HER potential, the LO phonon appears at 528.0 cm^−1^. This LO Raman mode is known to be induced by nonstoichiometric Ni‐O stretching in the NiO [50]. Notably, the LO peak disappears when the potential is returned to OCP after HER. This change indicates potential‐induced lattice distortion in NiO, suggesting structural instability under HER conditions. In contrast, the LO peak of NiO‐NiCo remains unchanged under the applied HER potentials, signifying the structural robustness of the NiO‐NiCo electrode, which is further supported by the SEM images (Figure 4h).

### 2.3 Applicability of NiO‐NiCo/NF to Industrial AWE

**FIGURE 5:**
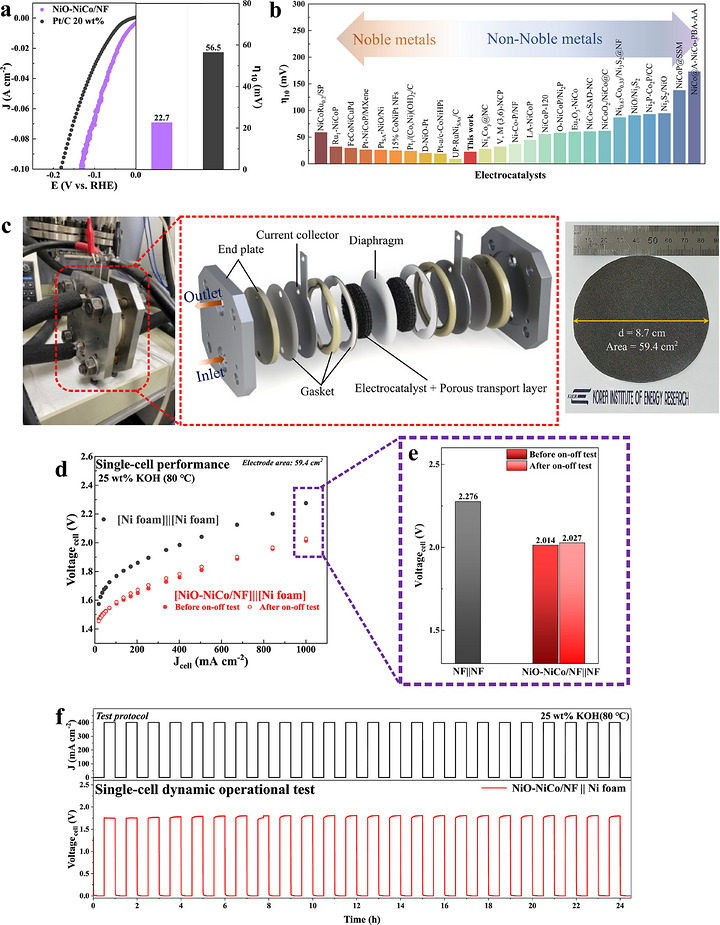
Practical applicability evaluation of NiO‐NiCo. (a) LSV curve and overpotentials at 10 and 100 mA cm^−2^ of NiO‐NiCo/NF. (b) Comparison of the overpotential at 10 mA cm^−2^ with those of recently reported catalysts. (c) Single‐cell photograph and configuration used in this study. (d) *I*–*V* polarization curves and (e) voltage at 1 A cm^−2^ of the NiO‐NiCo/NF||NF cell before and after the on‐off test and comparison with the results obtained for the NF||NF cell. (f) Profile of the NiO‐NiCo/NF||NF single cell recorded during the dynamic operation (on‐off) test.

Industrial AWE typically requires high efficiency and stability in highly alkaline electrolytes at current densities of 200–500 mA cm^−2^, with future industrial applications requiring low voltages even at high current densities of >1 A cm^−2^ [3, 69, 70]. However, Ni‐based electrodes face limitations in industrial KOH (25–30 wt.%) environments because of decomposition, surface structure changes, and leaching under polarization or shutdown (reverse current) conditions [71, 72, 73, 74, 75]. Given the excellent performance and durability of NiO‐NiCo, we evaluated its applicability to industrial AWE. To apply NF, which is widely used in AWE, NiO‐NiCo/NF was synthesized under the conditions used to prepare the NiO‐NiCo electrode, and its performance was evaluated in a three‐electrode cell in 1 M KOH at 25°C. NiO‐NiCo/NF exhibited overpotentials of 22.7 and 130.9 mV at 10 and 100 mA cm^−2^, respectively, which were 33.8 and 50.7 mV lower than those of Pt/C 20 wt.% (56.5 mV @ 10 mA cm^−2^, 181.6 mV @ 100 mA cm^−2^), demonstrating excellent performance. It outperformed previously reported NiO‐ and NiCo‐based catalysts and showed performance comparable to that of noble metal–containing NiO‐ and NiCo‐based electrodes (Figure 5a,b, Tables S3 and S4). Based on these results, a NiO‐NiCo electrode was synthesized on NF (diameter = 8.7 cm, area = 59.4 cm^2^) and integrated into a zero‐gap single‐cell (Figure 5c) that was pressurized from both sides using an end plate and comprised a current collector, porous transport layer, diaphragm (Zirfon UTP 500, Agfa, Belgium) enabling sealing with gaskets, and two electrodes. NiO‐NiCo/NF was used as the cathode, and NF was used as the anode (NiO‐NiCo/NF||NF configuration). A single cell featuring bare NF (NF||NF) as the cathode and anode was used for comparison. The experiment simulated a commercial AWE environment, with a 25 wt.% KOH solution circulated at a flow rate of 300 mL min^−1^ at 80°C. The *I*–*V* curves recorded under these conditions showed that at the industrially relevant current density of 1 A cm^−2^, the NF||NF cell required a high voltage of 2.276 V, whereas the NiO‐NiCo/NF||NF cell could be operated at 2.014 V, demonstrating an excellent performance despite the fact that only the HER catalyst was applied (Figure 5d,e). Moreover, the NiO‐NiCo/NF||NF cell outperformed the benchmark Pt/C||NF cell, which is evaluated in the 5 cm^2^ AWE single‐cell (Figure S26). For durability evaluation, an intermittent operation (on‐off) test was conducted under conditions capable of inducing decomposition and surface structure changes due to polarization and reverse current. The on (400 mA cm^−2^)/off (0 mA cm^−2^) cycling was performed at a 1 h interval for 24 h, and the effects of this cycling were probed by comparing the *I–V* curves recorded before and after the test (Figure 5f). During cycling, a minimal increase in voltage could appear as a sign of degradation; however, the starting voltage remained at the first‐cycle level upon turn‐off followed by turn‐on. This voltage increase was also observed for the catalyst‐free NF||NF configuration and was probably due to the influence of bubbles rather than electrode degradation (Figure S27a) [76, 77, 78, 79, 80]. The *I–V* curves recorded before and after cycling showed that NiO‐NiCo/NF||NF maintained its excellent performance, with the operation voltage at 1 A cm^−2^ after the test (2.027 V) increasing the pretest value by only 13 mV and thus demonstrating the commercialization potential of the NiO‐NiCo system. Conversely, the operation voltage of NF||NF at 1 A cm^−2^ after the test (2.33 V) increasing the pretest value by 54 mV (Figure S27b), which indicated that the stable structure of NiO‐NiCo/NF observed in the three‐electrode experiments was retained in the single cell. The synergistic effect of NiO‐NiCo enabled high performance and durability in the single cell as well. We further conducted the chronopotentiometry stability test of the NiO‐NiCo/NF||NF single cell, performed at 1 A cm^−2^ for 100 h (Figure S28a). It exhibited no noticeable voltage degradation even under the harsh operating condition of 1 A cm^−2^ for 100 h, demonstrating its robust stability and practical applicability for alkaline water electrolysis. Although NiO‐NiCo/NF||NF initially required 2.07 V to acquire 1 A cm^−2^, the cell voltage gradually decreased to 1.71 V at 1 A cm^−2^ after the electrochemical activation of the anode, satisfying the U.S. Department of Energy (DOE) 2026 technical target of 1.0 A cm^−2^ at 1.8 V for AWE (Figure S28b) [81]. The enhanced performance might originate from the electrochemical activation of the Ni foam anode. The Raman spectra confirmed that Ni(OH)_2_ is converted to NiOOH after the chronopotentiometry stability test, which is the catalytically active phase for the OER (Figure S29) [82]. Furthermore, TEM images of the NiO‐NiCo after 100 h of single‐cell operation confirmed the overall morphology of the NiO‐NiCo heterostructure (Figure S24b). In addition, XPS analysis demonstrated that the oxidation states of Ni and Co were preserved, although slight Co dissolution was observed during the stability test (Figure S25c,d). These results demonstrate the structural robustness of the NiO‐NiCo heterostructure even under practical operating conditions. These results demonstrate the potential of the NiO‐NiCo heterostructure for practical AWE, demonstrating that the transition‐metal‐based electrode can achieve industrially relevant performance while maintaining robust activity and cost‐effectiveness. Thus, the NiO‐NiCo electrodes were considered highly promising for practical alkaline water electrolysis applications due to their easy fabrication process and cost‐effectiveness resulting from the absence of noble metals.

## 3 Conclusion

A NiO‐NiCo electrode with high performance for the alkaline HER was fabricated by utilizing the insulating nature of NiO to form NiCo only in NiO‐free regions. The coexistence of NiO and NiCo on a single electrode resulted in a synergistic effect that targeted the high energy barrier of the Volmer step, a critical kinetic limitation of the alkaline HER. Physicochemical analysis confirmed the coexistence of NiO and NiCo. Electrochemical analysis, including comparisons with electrodes containing NiO and NiCo only, revealed that the synergistic interaction between these two materials markedly enhanced electrochemical activity. The NiCo‐only electrode exhibited a Tafel slope exceeding that of 120 mV dec^−1^, indicating slower kinetics dominated by the Volmer step. The incorporation of NiO effectively reduced the Tafel slope, which highlighted the acceleration of the Volmer step and HER kinetics enhancement due to the combined properties of the NiO‐NiCo configuration. EIS analysis further verified that the NiO–NiCo electrode exhibits reduced resistances for both the Volmer and Heyrovsky steps, reflecting the synergistic effect of the heterostructure. Durability testing demonstrated the high structural stability of the NiO–NiCo electrode, which is further supported by in situ Raman analysis showing that the NiO–NiCo electrode maintains its Raman spectral peak during HER, whereas NiO exhibits noticeable spectral changes indicative of lattice distortion under operating conditions. The NiO‐NiCo/NF electrode featured an overpotential of 22.7 mV at 10 mA cm^−2^, demonstrating a performance comparable with that of noble‐metal‐incorporated NiO‐ or NiCo‐based electrodes. In single‐cell tests, this electrode showed an operational voltage 262 mV lower than that observed for bare NF at 1 A cm^−2^, thus exhibiting a superior catalytic performance. This electrode also exhibited remarkable stability, showing an overpotential increase of only 13 mV after extensive on‐off testing, which indicated suitability for industrial applications.

## 4 Experimental Section

### 4.1 Chemicals and Materials

Nickel(II) nitrate hexahydrate (Ni(NO_3_)_2_∙6H_2_O, >99%), and Sulfuric acid solution (H_2_SO_4_, 30%) were purchased from Samchun Pure Chemical (Republic of Korea). Ammonium solution (NH_4_OH, 28%), Nickel(II) sulfate hexahydrate (NiSO_4_∙6H_2_O, >99%), Cobalt(II) sulfate heptahydrate (CoSO_4_∙6H_2_O, >99%), Ammonium sulfate ((NH_4_)_2_SO_4_, >99%), and Boric acid (H_3_BO_3_, >99%) were purchased from Junsei Chemical (Japan). 20 wt.% Pt/C was purchased from Alfa‐Aesar (United States). 5 wt.% Nafion ionomer, and Potassium hydroxide (KOH ≥ 85%, pellets) were purchased from Sigma–Aldrich (Germany). All chemicals were used without further purification.

### 4.2 Syntheses of Ni(OH)_2_/Ni and NiO/Ni

For the pretreatment process, a sandblasted coin‐shaped Ni plate (3.14 cm^2^, diameter = 2 cm, coin‐shaped) was ultrasonicated in deionized water for 60 min, rinsed with the deionized water, and dried in a vacuum chamber for 2 h. The pretreated Ni plate was placed at the bottom of a crystallizing dish containing a solution of Ni(NO_3_)_2_∙6H_2_O (5 mmol) in deionized water (20 mL) and NH_4_OH (80 mL) for hydrothermal synthesis. The dish was sealed and heated in an oven at 90°C for 8 h. The resulting Ni(OH)_2_/Ni was washed with deionized water and dried in a vacuum chamber at 40°C for overnight. The dried Ni(OH)_2_/Ni was then calcined in a furnace at 400°C in air with a heating rate of 2°C min^−1^ for 2 h to obtain NiO/Ni. After cooling to room‐temperature. To remove impurities other than NiO and after calcination, NiO/Ni was ultrasonicated in deionized water for 1 h, rinsed with the same, and dried in a vacuum chamber for 12 h.

### 4.3 Syntheses of NiO‐NiCo and NiCo/Ni

Electrodeposition was performed using a two‐electrode system in an electrolyte containing NiSO_4_∙6H_2_O (100 mM), CoSO_4_∙6H_2_O (60 mM), (NH_4_)_2_SO_4_ (25 mM), and H_3_BO_3_ (50 mM) at 25°C for 100 mA cm^−2^ (−7.4 V) for 125 s. The NiO/Ni and the Ni plate were used as the working electrode and counter electrode, respectively. The optimal electrodeposition conditions were identified by considering the effects of current density, voltage, time, and precursor concentration on the HER performance of NiO‐NiCo. For NiCo/Ni, electrodeposition was performed under the conditions used for NiO‐NiCo, except that the working electrode was a Ni plate and plating was conducted at a voltage of ‐7.4 V, while maintaining the same total passed charge.

### 4.4 Synthesis of NiO‐NiCo/NF

The NiO‐NiCo/NF was prepared using the same procedure as that used for NiO‐NiCo/Ni, with nickel foam (3.14 cm^2^, diameter = 2 cm, coin‐shaped, 110 PPI) employed as the substrate.

### 4.5 Characterization

Field‐emission scanning electron microscopy (SEM) imaging (Regulus 8220, Hitachi, Japan) was performed at an acceleration voltage of 5 kV. Energy‐dispersive X‐ray spectroscopy (EDS) analysis (ULTIM MAX, Oxford Instruments, United Kingdom) was carried out at 15 kV. Transmission electron microscopy (TEM) analysis was performed using a JEM‐F200 instrument (JEOL, Japan). Thermogravimetric analysis (TGA) was conducted by heating from room‐temperature to 800°C at a rate of 5°C min^−1^. X‐ray diffraction (XRD) patterns (Smart Lab High Resolution, Rigaku, Japan) were recorded using Cu *K*_α_ radiation (*λ* = 1.5406 Å) at 45 kV and 200 mA. X‐ray photoelectron spectroscopy (XPS) analysis was performed using a Thermo Fisher Scientific instrument at the Busan Center of the Korea Basic Science Institute (KBSI), and the binding energies were calibrated using the C 1s peak at 284.8 eV. Near‐edge X‐ray absorption fine structure (NEXAFS) measurements were performed at the 10A2 beamline of the Pohang Accelerator Laboratory (PAL), Republic of Korea, with spectra collected in the total electron yield mode. X‐ray absorption fine structure (XAFS) measurements were carried out at the 8C beamline of the PAL. The spectra were collected in fluorescence mode, with the storage ring operating at 3.0 GeV and a ring current of 300 mA. Energy calibration was conducted using reference metal foils. The raw XAS data were processed using the ATHENA program within the Demeter software package.

### 4.6 Electrochemical Analyses

Electrochemical analyses were conducted using a potentiostat/galvanostat/impedance analyzer (ZIVE SP2, Wonatech, Republic of Korea) and potentiostat (SP‐240, Biologic, France) in a three‐electrode system comprising working (NiO‐NiCo), reference (Hg/HgO; RE‐61AP, ALS, Japan), and counter (Ni plate) electrodes. Measurements were performed in 1 M KOH except for the durability test, which was conducted in 30 wt.% KOH. The Pt/C catalyst ink was prepared by dispersing 5 mg of commercial Pt/C (Alfa Aesar, 20 wt.%) with isopropyl alcohol (IPA) and deionized (DI) water (1:4, v/v), either with or without the addition of 40 µL of Nafion, followed by sonication for 20 min to obtain a homogeneous suspension. This ink was spray‐coated onto the carbon substrate. The Pt loading amount was controlled to 0.05 mg_Pt_ cm^−2^ for the half‐cell test and 0.20 mg_Pt_ cm^−2^ for the AWE single‐cell test. Performance evaluation was conducted using linear sweep voltammetry (LSV) at a sweep rate of 0.2 mV s^−1^ in a three‐electrode system at room‐temperature. All polarization curves were corrected with 100% *iR*‐compensation. Charge transfer resistances (*R*_ct_s) were calculated from electrochemical impedance spectra recorded in the frequency range of 100 kHz–0.1 Hz at a bias of −0.8 V vs. Hg/HgO. Non‐Faradaic double‐layer capacitance (*C*_dl_) was calculated from the electrochemically active surface areas (ECSAs), which were determined from cyclic voltammetry curves recorded at variable scan rates (20, 40, 60, 80, 100 mV s^−1^):

(4) $$C_{\mathrm{dl}}=Q/E=i/v$$

(5) $$\mathrm{ECSA}=C_{\mathrm{dl}}/C_{s}$$

where *C*_s_ = 0.04 mF cm^−2^ is the specific capacitance, *Q* (C) is the charge, *E* (V) is the potential, *i* (C s^−1^) is the current, and *v* (V s^−1^) is the scan rate. The electrode durability test was performed under dynamic operational conditions in 30 wt.% KOH at 25°C using on (−1.3 V) and off (0 V) cycles (1 min each, 600 cycles in total). LSV curves were recorded at 0.1 mV s^−1^ before and after cycling. Voltages were referenced to Hg/HgO and converted to the reversible hydrogen electrode (RHE) scale as

(6) $$E_{\mathrm{RHE}}\;\left(V\right)=E_{\mathrm{Hg}/\mathrm{HgO}}\;\left(V\right)+0.059\mathrm{pH}+0.098\;V$$

### 4.7 Synthesis of Au@SiO_2_ Shell‐Isolated Nanoparticles (SHINs)

The Au@SiO_2_ shell‐isolated nanoparticle (SHINs) were synthesized by a previously reported procedure. 2.7 mL of HAuCl_4_ (9.8 mM) was diluted to 200 mL of DI water in a round‐bottom flask and refluxed at 200°C under stirring. After the solution reached boiling, 1.4 mL of trisodium citrate solution (1 wt.%) was added. The mixture was then kept stirred and refluxed for 30 min, during which the solution color changed from yellow to maroon, indicating the formation of Au colloids (≈55 nm). The colloidal solution was stored in the dark at room‐temperature. For ligand exchange, 0.4 mL of (3‐aminopropyl)trimethoxysilane (APTMS) solution was added to 30 mL of Au suspension and stirred for 20 min. Subsequently, 3.2 mL of sodium silicate (0.54 wt.%, adjusted to pH 10.3 with diluted HCl) was then added to the solution and stirred at room‐temperature for 5 min. It is then transferred to a preheated water bath at 98°C to form a SiO_2_ shell of ≈2 nm thickness. The reaction was quenched by rapid cooling in an ice bath. The resulting nanoparticles were collected by centrifugation at a speed of 8000 rpm for 15 min and washed twice with DI water. Finally, the SHINs were dispersed in 200 µL of DI water and stored at 4°C for further use.

### 4.8 In Situ Raman Spectroscopic Analysis

In situ Raman measurements were carried out using a LabRAM HR Evolution (HORIBA, Japan) equipped with a 50× long working distance (LWD) objective. In situ Raman spectra were recorded at the Research Institute of Advanced Materials at Seoul National University (Republic of Korea), using a 532 nm excitation laser within a spectral range from 200 to 1300 cm^−^^1^. Wavenumber calibration was performed using a silicon reference sample (520.5 cm^−^^1^). Raman spectra were collected using a 600gr@500 nm. A custom‐designed electrochemical cell with a three‐electrode configuration was used for the in situ Raman measurements. The samples served as the working electrodes, while a platinum foil and a Hg/HgO electrode were used as the counter and reference electrodes, respectively. All measurements were conducted in 1 M KOH at room‐temperature. Raman spectra were recorded at open‐circuit potential (OCP) and under various applied potentials using a PGSTAT101 once the system reached a steady‐state current density

## Funding

This research was supported by the program of The Project of Technology Independence for Green Hydrogen through the National Research Foundation of Korea (NRF) funded by the Ministry of Science and ICT (RS‐2024‐00466616). This study was also supported by Project Code IBS‐R006‐A2 of the Republic of Korea.

## Conflicts of Interest

The authors declare no conflicts of interest.

## Supporting information




**Supporting File**: advs77112‐sup‐0001‐SuppMat.docx.

## Data Availability

The data that support the findings of this study are available from the corresponding author upon reasonable request.
