# Supplementary material for: Synergistic NiO‐NiCo Heterostructure Electrode for Highly Active and Durable Hydrogen Evolution in Industrial Alkaline Water Electrolysis
Source: Adv Sci (Weinh). 2026 Aug 15;13(54):e77112. doi: 10.1002/advs.77112 (PMC13477254; doi:10.1002/advs.77112)
Supplement: Supplementary file 1 [file ADVS-13-e77112-s001.docx]

Supporting Information

Synergistic NiO-NiCo heterostructure electrode for highly active and durable hydrogen evolution in industrial alkaline water electrolysis

Jinwoo Lee, Kyungbeen Yeom, Gaeun Hong, Yoojin Shin, Ji Seong Hyoung, Ji Eun Park, Heonjoong Lee, Taeyang Han, Yung-Eun Sung, Jeong Won Kang^*^, Kyoung-Soo Kang^*^, Hyunjoon Lee^*^

**Figure S1.** Scanning electron microscopy (SEM) images of (a) the Ni plate and (b) Ni(OH)_2_/Ni.

**Figure S2.** X-ray diffraction (XRD) patterns of the Ni plate and Ni(OH)_2_.

**Figure S3.** Thermogram of Ni(OH)_2_/Ni.

**Figure S4.** XRD patterns of Ni(OH)_2_/Ni and NiO/Ni.

**Figure S5.** SEM image of NiO/Ni.

**Figure S6.** Transmission electron microscopy (TEM) images of NiO/Ni.

**Figure S7.** SEM images and energy-dispersive X-ray spectroscopy (EDS) elemental mappings of (a) NiO/Ni and (b) NiCo-NiO.

**Figure S8.** TEM images of (a–c) NiO and (d–f) NiCo alloy in NiO-NiCo.

**Figure S9.** Scanning TEM (STEM)-EDS mappings of (a) NiCo and (b) NiO in NiO-NiCo. (c) Ni, O, and Co contents of NiCo and NiO in NiO-NiCo.

**Figure S10.** STEM image and EDS spectra of NiCo in NiO-NiCo.

**Figure S11.** (a) SEM, (b–e) TEM, and (f) STEM-EDS mapping images and (g) EDS spectrum of NiCo /Ni.

**Figure S12.** Schematic illustration of NiO-NiCo conducted electrodeposition (ED) for (a) 30 s, and (b) 600 s. SEM images of NiO-NiCo conducted ED for (c) 30 s, and (d) 600 s.

**Figure S13.** XRD patterns of NiO/Ni, NiCo/Ni, and NiO-NiCo.

**Figure S14.** Raman spectra of NiO/Ni, NiCo/Ni, and NiO-NiCo.

**Figure S15.** Raman spectra of the Ni plate, Ni(OH)_2_/Ni, and NiO/Ni.

**Figure S16.** Ni 2p spectra of the Ni plate and Ni(OH)_2_/Ni.

**Figure S17.** (a) Ni *L*-edge of NiO-NiCo, NiCo/Ni, and NiO/Ni. (b) Co *L*-edge of NiO-NiCo and NiCo/Ni.

**Figure S18.** (a) Equivalent circuit model, and (b) corresponding equivalent resistances.

**Figure S19.** Cyclic voltammograms of (a) NiCo/Ni, (b) NiO-NiCo, (c) NiO/Ni, and (d) Ni plate used to determine electrochemical surface areas.

**Figure S20.** SEM images of NiO/Ni before and after the on-off test. (b) SEM-EDS mapping of NiO/Ni after the on-off test.

**Figure S21.** SEM images of NiCo/Ni before and after the on-off test. (b) SEM-EDS mapping of NiCo/Ni after the on-off test.

**Figure S22.** SEM images of NiO-NiCo/Ni before and after the on-off test. (b) SEM-EDS mapping of NiO-NiCo/Ni after the on-off test.

**Figure S23.** (a) Chronopotentiometric stability test of NiO-NiCo at 100 mA cm^-2^ for 100 h. (b) LSV curves of NiO-NiCo before and after the 100 h chronopotentiometric stability test.

**Figure S24.** (a) TEM image of the NiO-NiCo after the half-cell long-term stability test at 100 mA cm^-2^ for 100 h (b) TEM image of the NiO-NiCo after the single-cell long-term stability test at 1 A cm^-2^ for 100 h

**Figure S25.** (a) Ni 2p, (b) Co 2p XPS spectra of NiO-NiCo after the half-cell long-term stability test at 100 mA cm^-2^ for 100 h. (c) Ni 2p, (d) Co 2p XPS spectra of NiO-NiCo after the single-cell long-term stability test at 1 A cm^-2^ for 100 h.

**Figure S26.** (a) AWE performance of Pt/C 20 wt%||Ni foam without ionomer. (b) AWE performance of Pt/C 20 wt%||Ni foam with ionomer.

**Figure S27.** (a) Dynamic operation (on-off) test profile of the Ni foam (NF)||NF single cell. (b) *I*–*V* polarization curves of the NF||NF cell recorded before and after the on-off test.

**Figure S28.** (a) Chronopotentiometric stability test of the NiO-NiCo/NF||NF single-cell performed at 1 A cm^-2^ for 100 h. (b) I–V polarization curves of the NiO-NiCo/NF||NF single-cell before and after the 100 h chronopotentiometric stability test.

**Figure S29.** Raman spectra of (a) Ni foam and (b) Ni foam after the single-cell long-term stability test at 1 A cm^-2^ for 100 h.

**Table S1.** Deconvolution result of Ni 2p XPS spectra for the NiO-NiCo, NiCo/Ni, and NiO/Ni.

| **NiO-NiCo** | **Binding energy of Ni 2p_3/2_ (eV)** | | | **Binding energy of Ni 2p_1/2_ (eV)** | | |
| --- | --- | --- | --- | --- | --- | --- |
|  | Ni^3+^ | Ni^2+^ | Ni^0^ | Ni^3+^ | Ni^2+^ | Ni^0^ |
|  | 856.5 | 855.2 |  | 874.3 | 872.7 |  |
|  | Ni^3+^/Ni^2+^=0.394 | | | | | |
|  | **FWHM of Ni 2p_3/2_ (eV)** | | | **FWHM of Ni 2p_3/2_ (eV)** | | |
|  | 2.77 | 2.174 |  | 2.751 | 2.368 |  |
| **NiCo/Ni** | **Binding energy of Ni 2p_3/2_ (eV)** | | | **Binding energy of Ni 2p_1/2_ (eV)** | | |
|  | Ni^3+^ | Ni^2+^ | Ni^0^ | Ni^3+^ | Ni^2+^ | Ni^0^ |
|  | 856.3 | 855.0 | 852.3 | 872.6 | 874.7 | 869.7 |
|  | Ni^3+^/Ni^2+^=0.338 | | | | | |
|  | **FWHM of Ni 2p_3/2_ (eV)** | | | **FWHM of Ni 2p_3/2_ (eV)** | | |
|  | 2.923 | 2.539 | 1.182 | 2.601 | 3.009 | 2.481 |
| **NiO/Ni** | **Binding energy of Ni 2p_3/2_ (eV)** | | | **Binding energy of Ni 2p_1/2_ (eV)** | | |
|  | Ni^3+^ | Ni^2+^ | Ni^0^ | Ni^3+^ | Ni^2+^ | Ni^0^ |
|  | 856.1 | 854.7 | 853.1 | 875.1 | 872.3 | 870.5 |
|  | Ni^3+^/Ni^2+^=0.439 | | | | | |
|  | **FWHM of Ni 2p_3/2_ (eV)** | | | **FWHM of Ni 2p_3/2_ (eV)** | | |
|  | 3.727 | 2.981 | 1.153 | 3.992 | 2.889 | 1.547 |

**Table S2.** Deconvolution result of Co 2p XPS spectra for the NiO-NiCo and NiCo/Ni.

| **NiO-NiCo** | **Binding energy of Co 2p_3/2_ (eV)** | | | **Binding energy of Co 2p_1/2_ (eV)** | |
| --- | --- | --- | --- | --- | --- |
|  | Co^3+^ | Co^2+^ | Co^0^ | Co^3+^ | Co^2+^ |
|  | 780.4 | 781.8 | - | 796.1 | 797.5 |
|  | Co^3+^/Co^2+^=1.53 | | | | |
|  | **FWHM of Co 2p_3/2_ (eV)** | | | **FWHM of Co 2p_3/2_ (eV)** | |
|  | 2.409 | 2.963 | - | 2.093 | 2.574 |
| **NiCo/Ni** | **Binding energy of Co 2p_3/2_ (eV)** | | | **Binding energy of Co 2p_1/2_ (eV)** | |
|  | Co^3+^ | Co^2+^ | Co^3+^ | Co^2+^ | Co^3+^ |
|  | 779.9 | 781.4 | 777.8 | 795.8 | 797.2 |
|  | Co^3+^/Co^2+^=1.39 | | | | |
|  | **FWHM of Co 2p_3/2_ (eV)** | | | **FWHM of Co 2p_3/2_ (eV)** | |
|  | 2.181 | 2.603 | 1.483 | 2.619 | 3.928 |

| Sample | Overpotential (mV) | | | Ref. |
| --- | --- | --- | --- | --- |
|  | **η _10 mA·cm_^-2^** | **η _100 mA·cm_^-2^** | |  |
| NiO-NiCo/NF | **22.7** | **130.9** | **This work** | |
| UP-RuNi_SAs_/C | 9 | - | *Nat. Commun*., 2024, **15**, 2218 | |
| Pt-a/c-CoNiHPi | 19 | - | *Small*., 2023, **19**, 2300368 | |
| D-NiO-Pt | 20 | - | *Adv. Energy Mater*., 2022, **12**, 2200434 | |
| Pt_1_/(Co,Ni)(OH)­_2_/C | 24 | 86 | *Energy Environ. Sci*., 2023, **16**, 1035 | |
| 15% CoNiPt NFs | 25 | - | *Adv. Funct. Mater*., 2023, **33**, 2303833 | |
| Pt_SA_-NiO/Ni | 26 | 85 | *Nat. Commun*., 2021, **12**, 3783 | |
| Pt-NiCoP/MXene | 26.5 | 84.8 | *Angew. Chem. Int. Ed*., 2024, **63**, e202401819 | |
| FeCoNiCuPd | 29.7 | - | *Appl. Catal. B: Environ*., 2022, **313**, 121472 | |
| Ru_1_-NiCoP | 32 | - | *Angew. Chem. Int. Ed*., 2023, **62**, e202308800 | |
| NiCoRu_0.2_/SP | 59 | - | *Appl. Catal. B: Environ*., 2023, **331**, 122710 | |

**Table S3.** Comparison of HER performance of NiO-NiCo with previously reported noble metal-containing NiO or NiCo-based electrocatalysts in 1.0 M KOH.

| Sample | Overpotential (mV) | | Ref. |
| --- | --- | --- | --- |
|  | **η _10 mA·cm_^-2^** | **η _100 mA·cm_^-2^** |  |
| NiO-NiCo/NF /NF | **22.7** | **130.9** | **This work** |
| C-Ni_1-x_O | 27 | - | *Nat. Commun*., 2020, **11**, 590 |
| Li_x_NiO/Ni | 36 | 111 | *J. Am. Chem. Soc*., 2020, **142**, 12613 |
| Ni-Co-P/NF | 37 | 176 | *Nat. Commun*., 2023, **14**, 1997 |
| LA-NiCoP | 45 | 131 | *Appl. Catal. B: Environ*., 2023, **339**, 123136 |
| NiCoP-120 | 56 | 247 | *Adv. Energy Mater*., 2023, **13**, 2300499 |
| O-NiCoP/Ni_2_P | 58 | - | *Appl. Catal. B: Environ*., 2021, **293**, 120196 |
| Eu_2_O_3_-NiCo | 60 | - | *Adv. Funct. Mater*., 2024, 2409324 |
| NiCo-SAD-NC | 61 | 189 | *Nat. Commun*., 2021, **12**, 6766 |
| MoO_3_/Ni–NiO | 62 | 347 | *Adv. Mater*., 2020, **32**, 2003414 |
| Ni_0.67_Co_0.33_/Ni_3_S_2_@NF | 87 | 203 | *Small*, 2022, **18**, 2106904 |
| NiO/Ni_3_S_2_ | 91 | - | *Chem. Eng. J*., 2023, **431**, 134137 |
| Ni_2_P-Co_2_P/CC | 93 | - | *Chem. Eng. J*., 2021, **424**, 130444 |
| Ni_3_S_2_/NiO | 95 | - | *Nat. Commun*., 2024, **15**, 5336 |
| Ni_58_Co_42_ | 162 | - | *Int. J. Hydrogen Energy*, 2017, **42**, 6637 |
| NiCo@A-NiCo-PBA-AA | 173 |  | *Adv. Funct. Mater.,* 2021, **31**, 2106835 |

**Table S4.** Comparison of HER performance of NiO-NiCo with previously reported NiO or NiCo-based electrocatalysts in 1.0 M KOH.
